# Prevalence and Outcomes of Myocarditis in Dengue-Infected Patients Admitted to a Tertiary Care Hospital of Low-Middle Income Country

**DOI:** 10.5334/gh.1129

**Published:** 2022-06-23

**Authors:** Abdul Baqi, Fazal ur Rehman, Pirbhat Shams Memon, Syed Furrukh Omair

**Affiliations:** 1 Aga Khan University Hospital, PK

**Keywords:** Dengue fever, dengue-myocarditis, heart failure, arrhythmias

## Abstract

**Background:**

Myocarditis is a challenging diagnosis due to the heterogeneity of clinical presentations. Myocarditis can present with a mildly raised cardiac enzyme to severe myocarditis leading to congestive heart failure, arrhythmias, cardiogenic shock, and death. It is a predictor of morbidity and mortality in dengue-infected patients. The exact prevalence of dengue myocarditis and its outcomes are unknown in Pakistan.

**Objectives:**

We aim to study the prevalence and association of myocarditis with the length of stay in the hospital and mortality of dengue-infected patients.

**Methods:**

A retrospective observational study done at a tertiary care hospital. We reviewed hospital record files of 1008 consecutive patients with dengue viral infection admitted from November 2018 to November 2019.

**Results:**

Out of 1008 dengue-infected patients, 55.4% of patients were older than 35 years and 68.4% were males. Hypertension (HTN) was the most common comorbid condition. The prevalence of myocarditis in hospitalized dengue-infected patients was 4.2%. All (100%) of dengue myocarditis patients had raised cardiac troponin I (cTn-I), 59.5% of patients had at least one electrocardiography (ECG) change, and 24% had reduced ejection fraction (EF) (defined as EF < 55%). On multivariable analysis, patients with raised cTn-I levels (adjusted odds ratios = 5.29; [95% confidence interval (CI): 2.16–12.96]) and abnormal echocardiography (ECHO) [aOR = 4.38; 95% CI: 1.26–15.27)] had a prolonged hospital stay (>3 days). Raised cTn-I levels (aOR = 8.2; [95% CI: 1.83–36.84]) was significantly associated with in-hospital mortality.

**Conclusions:**

Raised cTn-I is the predictor of length of stay and in-hospital mortality in dengue-infected patients. Atrial fibrillation, diabetes mellitus, hypertension, low serum bicarbonate, high serum creatinine, and any abnormality on echocardiography were associated with adverse outcomes in dengue-infected patients.

## Introduction

Viral illnesses are frequently complicated by cardiovascular manifestations such as arrhythmia, myocarditis, and myocardial injury. Dengue fever is known to be associated with myocarditis. The complex interplay of pro-inflammatory cytokines, T-cell activation, and release of vasoactive substances and vascular injury leads to increased vessel wall permeability and capillary leakage. Consequently, there is a reduction in effective preload and myocardial tissue edema. This leads to a variety of cardiac manifestations of dengue fever ranging from an asymptomatic elevation of cardiac enzymes to cardiogenic shock and, arrhythmias [1].

Cardiac manifestations of Dengue fever can present with a mildly raised cardiac enzyme to severe myocarditis leading to congestive heart failure, arrhythmias, cardiogenic shock, and death [2,3]. Cardiac complications during the illness carry significance in predicting length of stay (LOS) and in-hospital mortality [4].

The definitive diagnosis of myocarditis depends on an endomyocardial biopsy. However, the clinically suspected diagnosis is based on history, clinical examination, and biochemical and radiological profile. The European Society of Cardiology (ESC) 2013 consensus statement suggested the presence of at least one clinical and one diagnostic criterion for the diagnosis of clinically suspected myocarditis. Clinical criteria include acute new-onset, or worsening dyspnea, palpitations, and/or unexplained shock. The diagnostic criteria include ECG or Holter changes, raised cardiac biomarkers, functional and structural abnormalities on cardiac imaging, and tissue characterization on cardiac magnetic resonance (CMR) imaging [5,6].

The exact prevalence of dengue myocarditis is unknown. It is essential to recognize the burden of cardiac manifestations in dengue fever. There is a need for preparedness at the physician’s end for early recognition and prompt management for patients with dengue fever being complicated by cardiovascular manifestations. Additionally, determining prognostic factors is essential for the risk stratification of patients. With dengue fever continuing to be a major health care concern, more studies are needed to predict in-hospital outcomes and mortality. In this retrospective study, we looked at the prevalence and outcomes of myocarditis in hospitalized patients admitted with dengue fever.

## Material and Methods

It was a retrospective observational study done at the Aga Khan University hospital which is a 700-bedded multidisciplinary tertiary care hospital, located in the largest city of Pakistan. All patients (age more than 18 years) admitted with a clinical diagnosis of dengue viral infection from November 2018 to November 2019, were enrolled in the study. Data was retrieved from the electronic medical record system of the hospital. A positive dengue antigen (nonstructural protein-1, NS-1) or anti-dengue immunoglobulin M (IgM) assay (antibody-capture enzyme-linked immunosorbent assay, PanBio, Brisbane, Australia) was used as the inclusion criteria [7]. The platelet count on admission was used.

For all the patients, demographic and clinical information was recorded by reviewing individual medical records. Any patient, who during the hospital course, manifested with chest pain, shortness of breath, hemodynamic or electrical instability, new-onset heart failure, or shock underwent an ECG, cTn-I levels, and an ECHO to rule in the possibility of myocarditis. Patients were labeled as having suspected myocarditis as per the ESC criteria which required the presence of≥ 1 clinical feature and ≥1 diagnostic criteria in absence of known coronary artery disease (CAD) and other cardiac or extra-cardiac causes for the symptoms [5].

The 12 lead ECG was looked for any evidence of tachy-or bradyarrhythmia such as sinus arrest, atrioventricular block (AVB), bundle branch block, intraventricular conduction delay, atrial fibrillation, supraventricular tachycardia, frequent premature beats ventricular tachycardia or fibrillation and asystole, ST/T wave, reduced R-wave height, abnormal Q waves, or low voltage amplitude. The 12-lead ECG was interpreted by the cardiologist. The cutoff for the abnormal cardiac enzyme was cTn-I of more than 0.04 ng/ml. The echocardiographic features were looked for any evidence of systolic or diastolic dysfunction, pericardial effusion, ventricular dilatation, valvular regurgitation, or intra-cardiac thrombus. The troponin-I, CBC, and renal profiles were conducted using Advia Centaur, Sysmex Xn1000, and Advia 1800, respectively.

Patients with a history of myocarditis, heart failure, coronary artery disease, rheumatic heart disease, valvular heart disease, and congenital heart disease were excluded. Patients with acute and chronic toxicity, autoimmune diseases, renal failure, and pregnant women were also excluded, as well as, patients with an established dual infection such as dengue with malaria or dengue with Salmonella typhi.

## Statistical Analysis

All statistical analyses were performed using the Statistical Package for Social Sciences (SPSS) version 23 (IBM Corp., Armonk, NY). We reported frequencies and proportions for the categorical variables. The incidence of myocarditis was calculated using ESC 2013 diagnostic criteria for myocarditis [5]. We reported median and interquartile range (IQR) for the non-Gaussian distribution of the continuous variables. Pearson Chi-squared test or Fisher’s exact was used for assessing the frequency distribution and the relationship between co-variates and length of stay and mortality for categorical variables. We performed a univariate logistic regression analysis to determine the association between myocarditis and the independent effect of each significant predictor on length of stay and mortality. We considered a p-value of less than 0.05 for significant results. Finally, we conducted a multivariable logistic regression analysis to determine the association between myocarditis and LOS and in-hospital mortality while adjusting for all clinical characteristics. We presented the results of regression analysis by crude/unadjusted Odds ratio (OR) odds adjusted Odds ratio (aOR) with 95% Confidence Intervals (CIs).

## Ethical Consideration

The study was reviewed and approved by the ethical review committee of the Aga Khan University Hospital, Karachi, and was exempted from written informed consent.

## Results

### Clinical and biochemical characteristics of dengue-infected patients

Most patients were older than 35 years (55.4%) and males (68.4%). Hypertension was the most common co-morbidity (12.4%), followed by chronic kidney disease (12.2%), and diabetes mellitus (DM) (10.8%). Fifty-six point two percent of dengue-infected patients had platelets in between 40,000–150,000. Amongst 1008 patients enrolled, the overall prevalence of myocarditis in hospitalized dengue-infected patients was 4.16% (42 from 1008 patients). The overall mortality in dengue-infected patients was 1.6% (16 out of 1008 patients). Most of the patients (93%) were discharged home. The prevalence of cardiogenic shock was 9.5%. The in-hospital mortality of dengue myocarditis was 21.4%. The mean EF in dengue myocarditis patients was 50.8% (range from 15% to 55%) (Table 1). Diastolic dysfunction was present in 81.5% of patients. Right ventricle function was mildly reduced in 10% of dengue myocarditis patients, and 16% had pericardial effusion.

**Table 1:** Clinical and biochemical characteristics of dengue-infected patients (n = 1008). SCD = sudden cardiac death; PBNP = Pro brain natriuretic peptide; ECG = electrocardiography; ECHO = echocardiography; LAMA = left against medical advice; IQR = interquartile range.


CHARACTERISTICS OF DENGUE-INFECTED PATIENTS (N= 1008)	N (%)

Age (>35 years)	558 (55.4)

Gender (Male)	689 (68.4)

DM	109 (10.8)

Hypertension	125 (12.4)

Creatinine (>1.3 mg/dl)	114 (12.2)

**Platelets × 10^9^/L**	

>150,000	151 (15)

40,000 to 150,000	566 (56.2)

<40,000	291 (28.9)

**Discharge mode**	

Discharge	937 (93)

LAMA	55 (5.5)

In-hospital mortality	16 (1.6)

**PROFILE OF DENGUE MYOCARDITIS PATIENTS (N= 42)**	**N (%)**

Dyspnea	18 (42.9)

Palpitations	4 (9.5)

Shock (any)	12 (28.6)

Cardiogenic shock	4 (9.5)

Syncope	1 (2.4)

Aborted SCD	–

Fatigue	33 (78.6)

Fever	36 (85.7)

Troponin levels (>0.04)	42 (4.16)

PBNP pg/ml (Median IQR)	4609 (86–23300)

ECG abnormalities (at least one)	25 (59.5)

ECHO abnormalities (at least one)	10 (24)


### Characteristics of Dengue-infected patients stratified by in-hospital mortality

We found that dengue-infected patients with myocarditis were 6.07 (OR = 6.07; [95% CI: 1.75–21.05]) times more likely to die when compared with patients without myocarditis. Dengue infected patients who were diabetic were 6.78 times likely to die when compared to the non-diabetic patients (OR = 6.78; [95% CI: 2.47–18.61]). Similarly, hypertensive patients were 5.76 times likely to die when compared to non-hypertensive patients (OR = 5.76; [95% CI: 2.11–15.76]). Patients presenting with shock (OR = 4.43; 95% [CI 0.53 – 37]) and atrial fibrillation (OR = 4.05; [95% CI: 0.68 – 23.9s]) had higher odds of in-hospital mortality (Table 2).

**Table 2:** Socio-demographic and clinical characteristics of dengue patients by length of stay and in-hospital mortality. (n=1008). DM = diabetes mellitus; AVB = atrioventricular block; SCD = sudden cardiac death; ECG = electrocardiography; ECHO = echocardiography.


	LENGTH OF STAY					CRUDE			MORTALITY					CRUDE		

	≤3 DAYS		>3 DAYS		P-VALUE	OR	95%CI		YES		NO		P-VALUE	OR	95%CI	
			
	N	%	N	%					N	%	N	%				

**Age (Years)**																

≤35	359	45.6	91	41.2	0.24	1			6	37.5	444	44.8	0.56	1		
		
>35	428	54.4	130	58.8		1.19	0.88	1.62	10	62.5	548	55.2		1.35	0.48	3.74

**Gender**																

Male	549	69.8	140	63.3	0.07	1			10	62.5	679	68.4	0.61	1		
		
Female	238	30.2	81	36.7		1.34	0.97	1.83	6	37.5	313	31.6		1.3	0.45	3.61

**DM**																

No	710	90.2	189	85.5	0.04	1			9	56.3	890	89.7	<0.001	1		
		
Yes	77	9.8	32	14.5		1.56	1.01	2.43	7	43.8	102	10.3		6.78	2.47	18.61

**Hypertension**																

No	716	91	167	75.6	<0.001	1			9	56.3	874	88.1	<0.001	1		
		
Yes	71	9	54	24.4		3.26	2.2	4.83	7	43.8	118	11.9		5.76	2.11	15.76

**Dyspnea**																

No	6	50	18	60	0.55	1			2	22.2	22	66.7	0.02	1		
		
Yes	6	50	12	40		0.66	0.17	2.56	7	77.8	11	33.3		7	1.24	39.49

**Chest Pain**																

No	12	100	29	96.7	1	NA			9	100	32	97	1	NA		
	
Yes	0	0	1	3.3					0	0	1	3				

**Palpitations**																

No	9	75	29	96.7	0.06	1			7	77.8	31	93.9	0.2	1		
		
Yes	3	25	1	3.3		0.1	0.01	1.12	2	22.2	2	6.1		4.43	0.53	37.06

**Syncope**																

No	12	100	29	96.7	1	NA			9	100	32	97	1	NA		
	
Yes	0	0	1	3.3					0	0	1	3				

**Shock**																

No	9	75	29	96.7	0.06	1			7	77.8	31	93.9	0.2	1		
		
Yes	3	25	1	3.3		0.1	0.01	1.12	2	22.2	2	6.1		4.43	0.53	37.07

**Aborted SCD**																

No	12	100	30	100	NA	NA			9	100	33	100	NA	NA		
	
Yes	0	0	0	0					0	0	0	0				

**Platelets**																

>150,000	111	14.1	40	18.1		1			3	18.8	148	14.9		1		

40 to 150,000	441	56	125	56.6	0.22	0.78	0.52	1.18	5	31.3	561	56.6	0.09	0.44	0.1	1.86
		
<40,000	235	29.9	56	25.3		0.66	0.42	1.05	8	50	283	28.5		1.39	0.36	5.33

**Bicarbonate**																

≥22	623	82.8	139	65.9	<0.001	1			2	12.5	760	80.3	<0.001	1		
		
<22	129	17.2	72	34.1		2.5	1.77	3.52	14	87.5	187	19.7		28.45	6.41	126.26

**Creatinine**																

≤1.3	666	92	155	73.5	<0.001	1			4	25	817	88.9	<0.001	1		
		
>1.3	58	8	56	26.5		4.15	2.76	6.23	102	75	12	11.1		24.02	7.61	75.9

**Troponin-I**																

≤ 0.04	59	84.3	34	52.3	<0.001	1			4	30.8	89	73	0.003	1		
		
>0.04	11	15.7	31	47.7		4.89	2.18	10.96	9	69.2	33	27		6.07	1.75	21.05

**Echo findings**																

Normal	783	99.5	204	92.3	<0.001	1			13	81.3	974	98.2	0.004	NA		
	
Abnormal	4	0.5	17	7.7		16.31	5.43	49.01	3	18.8	18	1.8				

**AVB**																

No	10	100	29	100	NA	NA			8	100	31	100	NA	NA		
	
Yes	0	0	0	0					0	0	0	0				

**Atrial Fibrillation**																

No	9	90	23	79.3	0.65	1			5	62.5	27	87.1	0.14	1		
		
Yes	1	10	6	20.7		2.35	0.25	22.34	3	37.5	4	12.9		4.05	0.68	23.9

**Diastolic Dysfunction**																

None	2	40	2	8.7		NA			2	33.3	2	9.5		1		
	
Grade I	0	0	14	60.9	0.03				3	50	11	52.4	0.31	0.27	0.03	2.83
	
Grade II	3	60	6	26.1					1	16.7	8	38.1		0.12	0.007	2.17


A higher proportion of the patients who died were older than 35 years old (62.5%) when compared to 55.2% of the patients who survived. Patients with in-hospital mortality were more likely to be diabetics (p < 0.001) and hypertensive (p < 0.001) when compared to those who survived. Around 78% of the patients who died were dyspneic as opposed to 33.3% who survived (p-value: <0.02). A higher proportion of the patients (69.2%) who died had higher (>0.04) levels of cTn-I as opposed to 27% of the patients who survived. Patients with low serum bicarbonate on presentation had higher in-hospital mortality when compared to those with normal serum bicarbonate (87.5 vs. 11.1%, p < 0.001). Likewise, patients with in-hospital mortality had higher serum creatinine on presentation (75 vs. 25%, p < 0.001). Patients with higher in-hospital mortality had a higher percentage of abnormalities on ECHO (p = 0.004). Eighteen point eight percent of the patients who died were found to have abnormal findings on ECHO as opposed to 1.8% of the patients who survived (Table 2).

### Characteristics of Dengue-infected patients stratified by length of stay

Most patients with higher LOS were older than 35-year when compared with those with lesser LOS (58.8 vs. 54.4%). DM (14.5 vs. 9.8%, p 0.04) and Hypertension (24.4 vs. 9%, p < 0.001) were more common in patients with higher LOS when compared with patients with lower LOS. Patients with raised cTn-I were more likely to have higher LOS when compared with those with negative biomarkers (47.7 vs. 15.7%, p < 0.00). Dengue-infected patients with myocarditis were 4.89 (OR = 4.89; [95% CI: 2.18–10.96]) times more likely to have higher LOS. Dengue infected patient with DM (OR = 1.56; [95% CI: 1.01–2.43]) and Hypertension (OR = 3.26; [95% CI: 2.20–4.83]) had more likelihood of higher LOS (Table 2).

### Association of dengue-infected patients with a length of stay on multivariable analysis

Association of myocarditis with LOS was assessed by adjusting the results of multivariable analysis for age, gender, platelet count, echo findings, and DM. It was found that the association between raised cTn-I (aOR = 5.29; [95% I:2.16–12.96]) and any echocardiographic abnormality [aOR = 4.38; 95% CI: 1.26–15.27)] with patient’s LOS beyond three days persisted in the adjusted model and became stronger relative to the bivariate analysis as shown in Table 3. However, the association of age, gender, DM, and thrombocytopenia with increased LOS disappeared in the final adjusted model after controlling for potential confounders (Table 3).

**Table 3:** Association of myocarditis with morbidity and mortality (n = 1008). aOR = adjusted odds ratio; CI = confidence interval.


	LENGTH OF STAY > 3 DAYS			MORTALITY		
	
	AOR	95% CI		AOR	95% CI	

**Age (Years)**						

≤35	1			1		

>35	0.66	0.24	1.81	0.22	0.04	1.34

**Gender**						

Male	1			1		

Female	1.24	0.55	2.77	2.82	0.68	11.67

**Troponin levels**						

≤0.04	1			1		

>0.04	5.29	2.16	12.96	8.2	1.83	36.84

**Diabetes Mellitus**						

No	1			1		

Yes	0.67	0.27	1.68	1.48	0.34	6.49

**Platelets**						

>150,000	1			1		

40 to 150,000	0.41	0.13	1.32	0.28	0.04	1.95

<40,000	0.37	0.1	1.31	0.63	0.09	4.33

**Echo Findings**						

Normal	1			1		

Any cardiac abnormality	4.38	1.26	15.27	2.24	0.42	11.97


### Association of Dengue-infected patients with mortality on multivariable analysis

After adjusting the results of multivariable analysis for age, gender, platelet count, echo findings, and DM, the result demonstrated a statistically significant association of raised cTn-I (aOR = 8.2; [95% CI: 1.83–36.84]), echocardiographic abnormality (aOR = 2.24; [95% CI: 0.42–11.97]) and female gender (aOR = 2.82; [95% CI (0.68–11.67]) with increased in-hospital mortality as shown in Table 3. Age and thrombocytopenia were not significantly associated with mortality (Table 3).

## Discussion

There are several predictors of outcomes in dengue fever, such as extremes of age, hypertension, diabetes mellitus, nutrition status, and superadded infections [8]. In addition, altered mental status, and dyspnea at rest have been defined as independent predictors of outcomes in dengue illness [9]. The exact impact of abnormal cardiac biomarkers and ECG (myocarditis) on the prognosis of dengue illness is variable. In our study, we found that patients with myocarditis had higher odds of in-hospital mortality and increased LOS. Raised cTn-I and any echocardiographic abnormality were associated with increased in-hospital mortality.

Our study showed a myocarditis prevalence of 4.2%. Majumdar et al. estimated the prevalence of myocarditis in 300 dengue patients during an epidemic and found it to be 20% [10]. During China’s dengue outbreak in 2014, the estimated prevalence of dengue myocarditis in hospitalized patients was 11.28% (201 out of 1782 patients). The prevalence was higher in non-severe or severe dengue illness with warning signs in comparison to non-severe dengue illness without warning signs (46.66% vs. 9.72%).

In our study, 100% of dengue myocarditis patients had raised cTn-I, 59.5% of patients had at least one ECG change, and 24% had reduced EF (defined as EF < 55%). In the characterization of 201 dengue myocarditis patients in China, Yingying et al. demonstrated that 24% had positive cardiac biomarkers, 8.63% had echocardiographic changes, 8.46% had ECG changes, 22.58% had both positive cardiac biomarkers and echocardiographic changes, and 21% had both positive cardiac biomarkers and ECG changes [4]. Likewise, in a pediatric dengue outbreak in Indonesia, of 39 myocarditis patients, 24% had raised cTn-I, 70% had raised creatinine kinase – MB (CKMB) and 44% had at least one ECG change [11].

ECG abnormalities can be frequently encountered in viral fever. This can include a variety of arrhythmias such as heart block, ectopic beats, and incessant tachyarrhythmias [12,13,14]. These are usually transient. Supraventricular tachycardias (including atrial fibrillation) are frequently encountered because of underlying myocardial injury and systemic inflammatory response. We had a very low prevalence of ECG abnormalities overall (0.7% of all dengue infected patients) but ECG change was the second most common diagnostic criterion in 42 dengue myocarditis patients. Intraventricular conduction delay (IVCD) was the most common ECG abnormality encountered (26%) and atrial fibrillation was the most common tachyarrhythmia encountered. Approximately 31% of dengue myocarditis patients had sinus tachycardia on presentation (13 out of 42), only 3 patients had sinus bradycardia, 14% (6 out of 42) had atrial fibrillation, 17% (7 out of 42) had ST-T changes, 26% (11 out of 42) had IVCD and 19% (8 out of 42) had poor-R-wave progression (PRWP) on ECG. Arora et al. showed that in a cohort of 120 patients with dengue fever, raised cardiac markers were noted in 33.3% and 26.7% (for CK-MB and Troponin-I respectively). The prevalence of myocarditis was 37.5% and there was a positive correlation with dengue illness severity as per World Health Organization (WHO) grading. Other cardiac manifestations included: arrhythmias in 5% of patients with atrioventricular block being the most common entity (66.7%) [15]. However, in another cohort of 81 patients, Miranda et al. showed that patients with raised cardiac biomarkers had no correlation with WHO disease severity, duration of symptoms, or prevalence of secondary infections but had higher leukocyte and platelet counts and higher C-reactive protein levels [2].

In our study, fever was the most common clinical presentation in dengue myocarditis patients (85.7%), followed by fatigue (78.6%), dyspnea (42.9%), and hypotension (28.6%), palpitation (9.5%) and syncope (2.4%). Cardiogenic shock was present in 9.5% of our myocarditis patients. In a cohort of 128 dengue-infected patients admitted to a multidisciplinary hospital in Pakistan, the prevalence of dengue myocarditis was 18.75% (24 out of 128 dengue patients). In this cohort of dengue myocarditis patients, fever (100%), dyspnea (100%), and fatigue (100%) were the most common clinical features on presentation. All patients had raised cardiac biomarkers, globally reduced ejection fraction, and hypotension. The shock was classified as cardiogenic in 19.67% of patients. This cohort carried a very high in-hospital mortality (83.3%). Sinus tachycardia (87.5%) was the most common ECG finding followed by atrial fibrillation (8.3%) and ventricular ectopic (4.1%) [16].

In our study, 24% of dengue myocarditis patients had reduced EF (defined as EF less than 55%). The mean EF was 50.8% (range from 15% to 55%). Sengupta et al. studied 2D speckle tracking echocardiograms of patients with dengue hemorrhagic fever (DHF) and compared them to control. It was found that patients with DHF had lower EF and significantly attenuated subendocardial peak longitudinal strain. Lower strain predicted LOS of DHF patients [17]. Additionally, the Tei index has also been evaluated in dengue myocarditis and it is of value in detecting patients with asymptomatic myocarditis not otherwise detected by conventional EF measurements [18].

Prognostic outcomes in myocarditis due to any reason include gender, cardiac enzymes level, NYHA class, and creatinine clearance or incidence of acute kidney injury [19]. Dengue myocarditis is a rare but possibly fatal condition. It is unknown what are the prognostic markers for this disease entity. Our study showed that low serum bicarbonate, higher serum creatinine, any echocardiographic abnormalities, diabetes mellitus, and hypertension were associated with adverse prognosis. In the Chinese cohort of dengue myocarditis, there was a higher incidence of arrhythmias (Supraventricular tachycardia and atrial fibrillation) and heart failure in those presenting with warning signs. Myocarditis was a predictor of length of stay in this cohort. There was a trend toward a higher incidence of shock and deranged liver enzymes in those with myocarditis [4].

Our study is not free of limitations. It is a retrospective observational study, conducted in a single center and the sample size is small which may not represent the whole Pakistani population. There is no long-term follow-up. We suggest prospective randomized studies to predict the long-term outcomes of Dengue myocarditis patients in our region.

## Conclusion

Dengue fever complicated by myocarditis has prognostic implications. Physicians should be cognizant of the chances of higher mortality and prolonged hospital stay in patients presenting with dengue fever and any degree of myocarditis. This further helps identify patients who would require telemetry monitoring and in-hospital cardiology consultation.

## Additional File

The additional file for this article can be found as follows:

10.5334/gh.1129.s1 Dataset.
